# Supplementary material for: Identification of photoperiod-induced specific miRNAs in the adrenal glands of Sunite sheep (Ovis aries)
Source: Front Vet Sci. 2022 Jul 22;9:888207. doi: 10.3389/fvets.2022.888207 (PMC9354845; doi:10.3389/fvets.2022.888207)
Supplement: Supplementary file 1 [file Data_Sheet_1.ZIP › Supplementary file/miRDeep2 file.zip!/miRDeep2 file/Know_Structure/SPLP42A1/oar-mir-323a.pdf]

miRBase precursor : oar-mir-323a
Total read count : 2408
oar-miR-323a-5p read count : 15
oar-miR-323a-3p read count : 2390
remaining reads : 3

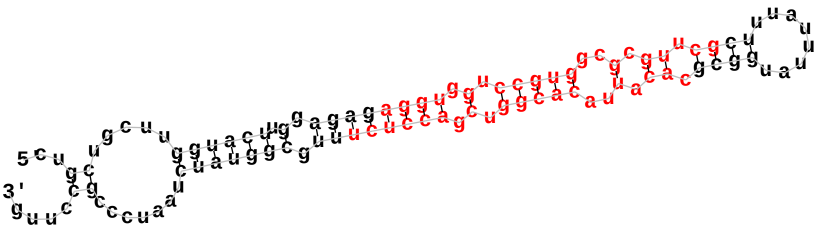

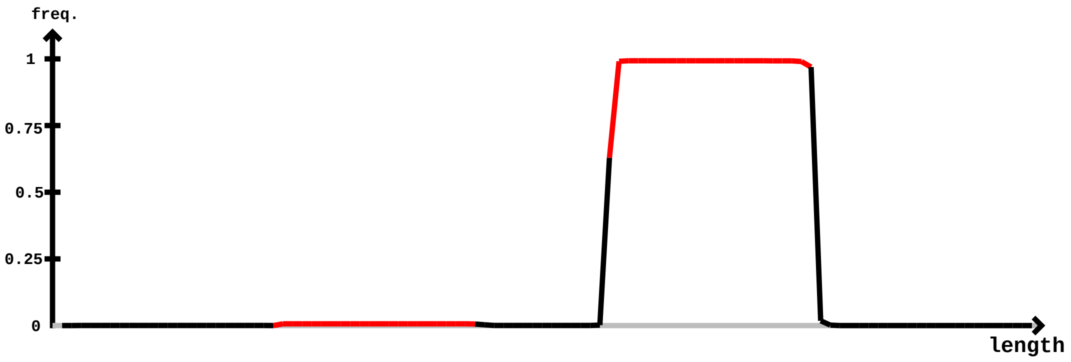

oar-miR-323a-5p

oar-miR-323a-3p

```
5'- cugcugcuugguacuuggagagaggugguccguggcgcguucgcuuuauuuauggcgcacauuacacggucgaccucuuugcgguaucuaaucccgccuug   -3'    exp
    .........((((((.(.((((((((.(.(((((..(.((.((((........)))).)).)..))))).).)))))))).)))))))..............     reads      mm         sample
    .ugcugcuugguacuuggaga.................................................................................     1          0          seq
    ......................agguggucc guggcgcguuc...........................................................     2          0          seq
    ......................aggugguccguggcgcguucg..........................................................     7          0          seq
    ......................aggugguccguggcgcguucgc.........................................................     6          0          seq
    ......................aggugguccguggcgcguucgcuuuauu....................................................     1          0          seq
    ...................................................uauggcgcacauuacacggucga............................     1          0          seq
    ........................................................cgcacauuacacggucgaccucu.......................     3          0          seq
    .........................................................gcacauuacacggucgaccu.........................     2          0          seq
    .........................................................gcacauuacacggucgaccuc........................     32         0          seq
    .........................................................gcacauuacacggucgaccucu.......................     1456       0          seq
    .........................................................gcacauuacacggucgaccucuu......................     19         0          seq
    .........................................................gcacauuacacggucgaccucuuu.....................     2          0          seq
    ..........................................................cacauuacacggucgaccu.........................     4          0          seq
    ..........................................................cacauuacacggucgaccuc........................     18         0          seq
    ..........................................................cacauuacacggucgaccucu.......................     828        0          seq
    ..........................................................cacauuacacggucgaccucuu......................     20         0          seq
    ..........................................................cacauuacacggucgaccucuuu.....................     1          0          seq
    ...........................................................acauuacacggucgaccucu.......................     4          0          seq
    ...........................................................acauuacacggucgaccucuuu.....................     1          0          seq
```
